# Case report: Extracorporeal life support as a successful bridge to recovery in an incident case of pulmonary arterial hypertension

**DOI:** 10.3389/fmed.2024.1283065

**Published:** 2024-02-06

**Authors:** Benjamin Pequignot, Ari Chaouat, François Chabot, Bruno Levy, Simon Valentin

**Affiliations:** ^1^Université de Lorraine, Service de Médecine Intensive et Réanimation, Hôpital Brabois, CHRU Nancy, Vandoeuvre les Nancy, France; ^2^Université de Lorraine, Faculté de Médecine de Nancy, Inserm UMR_S1116, Vandœuvre-Lès-Nancy, France; ^3^Université de Lorraine, CHRU-Nancy, Pôle des spécialités médicales/département de pneumologie, Nancy, France; ^4^IADI, Université de Lorraine, INSERM U1254, Nancy, France

**Keywords:** PAH, ECMO, pulmonary hypertension, prostacyclin, ECLS, cardiac failure

## Abstract

Pulmonary arterial hypertension (PAH) is characterized by a progressive increase in pulmonary vascular resistance (PVR) due to vascular remodeling of the small pulmonary arteries. In advanced RV failure or severe hypoxemia, extra corporeal life support (ECLS) is now to be considered, with the objective to bridge patients back to their baseline clinical state while waiting or right after lung transplantation, or bridge to pharmacological optimization of PAH (i.e., bridge to recovery). We describe herein a case of a 30-year-old woman (*gravida* 6, *para* 6) with an incident case of heritable PAH revealed by refractory hypoxemia. Despite the use of mechanical ventilation and fluid optimization, the patient remained profoundly hypoxemic. ECLS was then initiated to avoid tissue hypoxia. The mechanical option chosen was peripheral femoro-femoral venoarterial extracorporeal membrane oxygen (VA-ECMO), percutaneously implanted. Due to the absence of evidence of chronic respiratory disease or chronic thromboembolic pulmonary hypertension, this severe pre-capillary pulmonary hypertension was attributed to PAH. Therefore, epoprostenol infusion and an association of oral treatments (bosentan and tadalafil) were administered. A dramatic improvement was observed, allowing decannulation 7 days after the initiation of pharmacological treatment. After 29 days, the patient was discharged from the hospital with epoprostenol, bosentan, and tadalafil. The assessment has been completed by positive research on mutations (*c.741C > G, p.Tyr247*) corresponding to a loss of function of the bone morphogenetic protein receptor 2 (*BMPR2*) gene. The final diagnosis was heritable PAH. The use of ECLS has been well demonstrated in patients with PAH complicated by acute RV failure or refractory hypoxemia in the “bridge-to-transplantation” strategy. Only a few reports have described the use of ECLS as a “bridge-to-recovery” with PAH drugs in untreated or undertreated PAH patients, but none has described such a rapid improvement with resolution of refractory hypoxemia. More studies are needed to assess the benefits and limitations of the “bridge-to-recovery” strategy and to identify the patients most likely to benefit from it.

## Introduction

Pulmonary arterial hypertension (PAH) is characterized by a progressive increase in pulmonary vascular resistance (PVR) due to vascular remodeling of the small pulmonary arteries (1). The course of the disease can be complicated by acute right ventricular (RV) failure, which is associated with a 30–40% mortality rate in the intensive care unit (ICU) (2). Patent *foramen ovale* (PFO) is frequently reported in this situation and creates a right-to-left (RL) shunt, reducing RV workload but leading to hypoxemia (3). Management of advanced RV dysfunction relies on the correction of a possible triggering factor, hemodynamic optimization (fluid volume management and pharmacological support), and the reduction of RV afterload using PAH drugs. In advanced RV failure or severe hypoxemia, extra corporeal life support (ECLS) is now to be considered, with the objective of bridging patients back to their baseline clinical state in a few situations: while waiting or right after lung transplantation, or as a bridge to pharmacological optimization of PAH (i.e., a bridge to recovery) (4). Reports of ECLS use in patients with acute RV failure or facing severe hypoxemia as a bridge to the initiation of PAH drugs in incident cases are very limited (5–7). We report here a patient with previously undiagnosed PAH, revealed by severe hypoxemia, and successfully bridged to recovery due to PAH therapy after the implantation of ECLS.

## Case report

### Presentation

**Figure 1:**
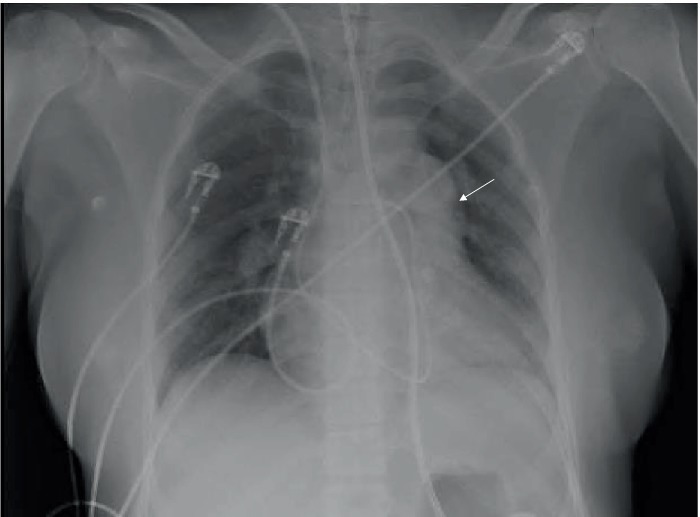
Chest radiography performed after intensive care unit admission. A large left central pulmonary artery with obliteration of the aortopulmonary window (white arrow).

**Figure 2:**
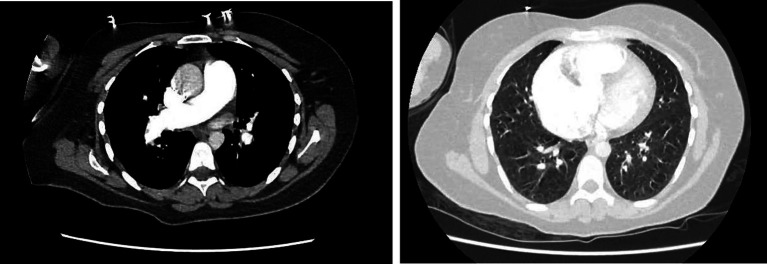
Initial computed tomography of the chest. Absence of parenchymal abnormalities. Enlargement of the trunk of the pulmonary artery (35 mm). There was no pulmonary embolism. Both panels are axial views.

**Figure 3:**
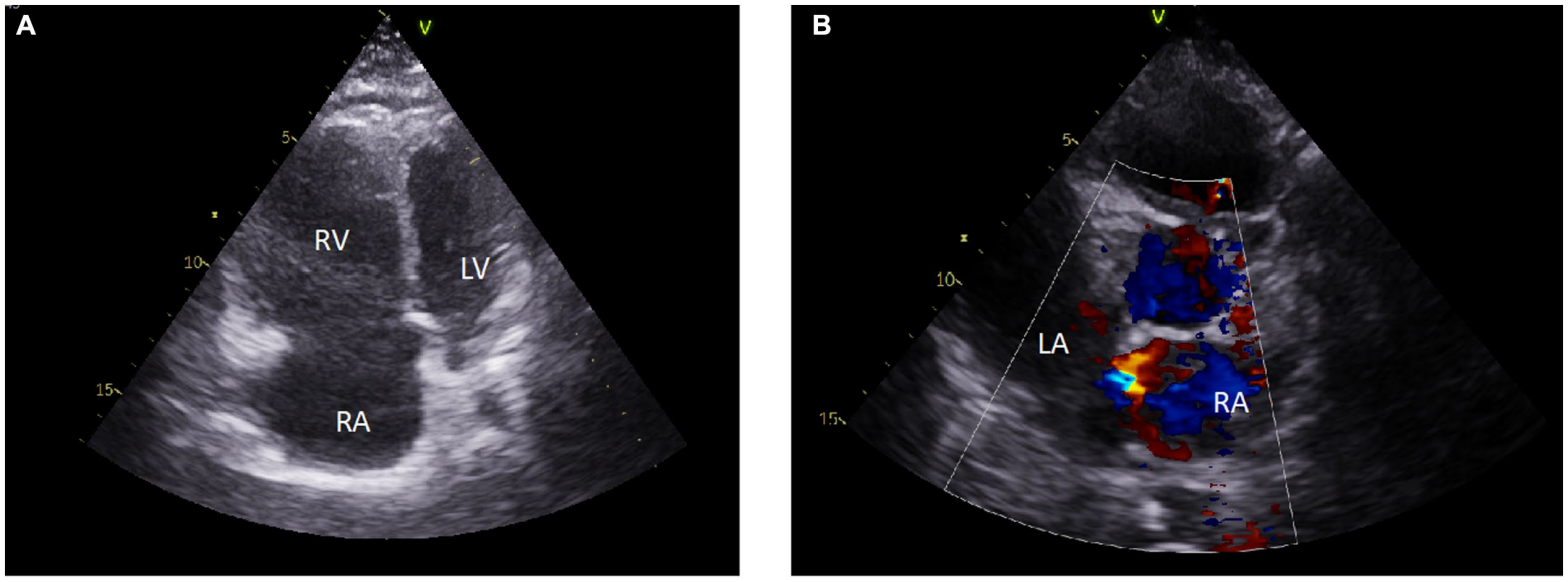
Initial transthoracic echocardiography. Echocardiograms showed an enlarged right ventricular cavity, a dilated right atrial cavity, and a small left ventricular cavity with flattening of the interventricular septum **(A)**. There was evidence of patent *foramen ovale* **(B)**. RA, right atrial cavity; RV, right ventricular cavity; LV, left ventricular cavity.

A 30-year-old woman (*gravida* 6, *para* 6, last pregnancy 36 months before admission) with no prior medical history experienced progressively worsening shortness of breath during exertion for 1 month before being hospitalized. She was admitted to the emergency room due to a marked increase in shortness of breath, even while at rest. Temperature was 37.5°C, blood pressure: 104/82 mmHg, heart rate: 112 beats per minute, respiratory rate: 17 breaths per minute, and pulse oxygen saturation of 92% while breathing under a high-flow nasal cannula (HFNC) with a fraction of inspired oxygen (F_i_O_2_) 1. On physical examination, we noted pulsating jugular veins, tachycardia with a regular rhythm, increased loudness of the second heart sound, and no abnormalities on pulmonary auscultation. The patient reported severe shortness of breath. No organomegaly and no edema were found. A complete blood count revealed a white cell count of 14,200 cells/mm^3^ (56.2% lymphocytes, 37.2% neutrophils, 5% monocytes, 0.8% eosinophils, and 0.8% basophils), a hemoglobin level of 14.2 g/dL, and a platelet count of 254,000 cells/mm^3^. Kidney and liver blood tests were within the normal range. N-terminal prohormone brain natriuretic peptide (NT-pro BNP) level was 1,328 pg./mL. Arterial blood gas (ABG) under HFNC at FiO_2_ 1 revealed pH: 7.45, PaCO_2_: 25 mmHg, PaO_2_: 66 mmHg, HCO_3_^−^: 19 mmol/L, SaO_2_: 92%, lactate 0.7 mmol.L^−1^. The shunt ratio estimate was 27%, according to the following formula: (P_A_O_2_ − PaO_2_)/(P_A_O_2_ − PaO_2_ + 1,670) (8). Chest radiography found an increased cardio-thoracic index and a protrusion of the left middle arch (Figure 1). Computed tomographic (CT) angiography of the chest revealed no parenchymal abnormalities, no pulmonary embolism, but a dilatation of the pulmonary artery trunk to 35 mm and a ratio of pulmonary artery diameter to aortic diameter of 1.1. There was no pleuropericardial effusion (Figure 2). The electrocardiogram showed sinus tachycardia with right-axis deviation and a high P-wave in leads II, III, and V3–V5. A transthoracic echocardiogram (TTE) showed a left ventricle with an ejection fraction of 55% and a normal left morphology. There was severe dilatation of the RV with reduced systolic function; the systolic excursion of the tricuspid annular plane was 16 mm, and the RV S-wave was 10 cm/s. The estimated systolic pulmonary artery pressure was 93 mmHg. Transthoracic contrast echocardiography showed early positive contrast and confirmed PFO (Figure 3). There was no evidence for congenital heart disease. The patient was transferred to the ICU for the management of severe hypoxemia.

In the ICU, right heart catheterization was performed and confirmed severe pre-capillary pulmonary hypertension with a mean pulmonary artery pressure (mPAP) of 65 mmHg, right atrial pressure (RAP) of 15 mmHg, pulmonary arterial wedge pressure (PAWP) of 14 mmHg, cardiac index of 1.7 L/min/m^2^, and a PVR of 18 *Woods units (WU)* (Table 1). Faced with hypoxemic respiratory failure, mechanical ventilation (MV) was initiated. MV was optimized with high FiO_2_ and PEEP level titration using alveolar opening pressure and personalized tidal volume in volume control mode. High doses of diuretics were introduced, guided by the adapted monitoring. Despite this, the patient remained profoundly hypoxemic (ABG with FiO_2_: 1: pH: 7.36, PaCO_2_: 44 mmHg, PaO_2_: 56 mmHg, HCO_3_^−^: 20 mmol/L, SaO_2_: 87%, lactate 2.1 mmol.L^−1^). ECLS was then initiated to avoid tissue hypoxia. The mechanical option chosen was peripheral femoro-femoral venoarterial extracorporeal membrane oxygen (VA-ECMO), percutaneously implanted with arterial cannula of 15 Fr and venous cannula of 19 Fr. The ECMO flow was set at 3.5 L/min, the sweep was set at 2.0 L/min and the fraction of oxygen in the membrane was set at 0.45. After VA-ECMO implantation, lung-protective ventilator strategies were used (i.e., targeting at low tidal volume = *V*_T_ of 6–8 mL/Kg predicted body weight (PBW), low plateau pressure = *P*_PLAT_ < 20 cmH_2_O_,_ driving pressure = Δ*P* < 13 cmH_2_O, and low positive end-expiratory pressure (PEEP) < 7 cmH_2_O).

**Table 1:** Clinical, biological, and hemodynamical course of the patient.

	ICU admission	Discharge from ICU	6 months follow-up	2 years follow-up
Clinical signs				
NYHA functional class	IV	III	II	II
RHF signs	No	No	No	No
NT-proBNP (pg/mL)	1,328	293	296	489
Transthoracic echocardiography				
Estimated sPAP (mmHg)	93		60	56
RV dilatation	Yes		Yes	Yes
RV/LV ratio	2		1.3	1.2
TAPSE (mm)	16		18	20
RV S-wave (cm/s)	10		13	14
Pericardial effusion	No		No	No
PFO	Yes		No	No
Right heart catheterization				
mPAP (mmHg)	65		41	54
sPAP (mmHg)	100		65	89
dPAP (mmHg)	45		25	54
PAWP (mmHg)	14		5	6
RAP (mmHg)	15		2	5
PVR (WU)	18		7,9	8.8
CI (mL/min/m^2^)	1.7		2.9	3.4
6MWD distance (m)		339	480	487
Vasoactive molecules				
Epoprostenol (ng/kg/min)		14	16	20
Bosentan (mg)		250	250	250
Tadalafil (mg)		40	40	40

6MWD, 6-min walk distance; CI, cardiac index; LV, left ventricle; NT-pro-BNP, N-terminal prohormone of the Brain Natriuretic peptide; NYHA, New York Heart Association; PAWP, pulmonary artery wedge pressure; PFO, Patten foramen ovale; PVR, pulmonary vascular resistance; RAP, right atrial pressure; RHF, right heart failure; RV, right ventricle; s/d/mPAP, systolic/diastolic/mean pulmonary arterial pressure; TAPSE, tricuspid annular plane systolic excursion.

### Management

Due to the absence of evidence for chronic respiratory disease or chronic thromboembolic pulmonary hypertension based on medical history and chest CT angiography, this severe pre-capillary pulmonary hypertension was attributed to PAH. Therefore, a combination of PAH drugs was promptly initiated. Continuous intravenous epoprostenol infusion (basic flow rate at 4 ng/kg/min, gradually increased every 12 h to a dose of 10 ng/kg/min) and an association of oral treatments (bosentan and tadalafil) were administered. A dramatic improvement was observed under PAH drugs, allowing decannulation 7 days after the initiation of pharmacological treatment. The patient was discharged from intensive care and transferred to a hospital ward. Pulmonary function tests were performed and showed no obstructive or restrictive pattern, but a decrease in diffusing capacity for carbon monoxide to 56% of what was predicted. The ventilation-perfusion scan did not find any mismatches. After 29 days, the patient was discharged from the hospital with epoprostenol (flow rate at 16 ng/kg/min, gradually increasing), bosentan, and tadalafil. The World Health Organization (WHO) functional class was III, NT-proBNP: 293 pg./mL, 6-min walk test distance (6MWD) was 339 meters (Table 1) (9). ABG with F_I_O_2_ 0.21 was pH: 7.43, PaCO_2_: 30 mmHg, PaO_2_: 72 mmHg, SaO_2_: 95%. The assessment has been completed by the positive research of mutation (*c.741C > G, p.Tyr247*) corresponding to loss of function of the bone morphogenetic protein receptor 2 (*BMPR2*) gene, confirmed on two separate samples. The final diagnosis was heritable PAH.

### Follow-up

Clinical, biological, and hemodynamical data after 2 years of follow-up are presented in Table 1 and indicate a low-risk status.

## Discussion

We present herein an incident case of heritable PAH revealed by an acute hypoxemic respiratory failure explained by a large PFO, which has a dramatic hemodynamic and clinical improvement under PAH drugs, supported by ECLS.

### Mechanical support for the failing RV or severe hypoxemia

The use of ECLS has been well demonstrated in patients with pulmonary hypertension of WHO Group 1 complicated by an acute RV failure or refractory hypoxemia in the “bridge-to-transplantation” strategy (4). Only a few reports have described the use of ECLS as a “bridge-to-recovery” with PAH drugs in untreated or undertreated PAH patients (5–7). There are various devices to assist RV or to manage severe hypoxemia, including direct or indirect RV bypass, as well as intracorporeal (Impella) and extracorporeal (right ventricular assist device or ECMO) options (10). The primary mechanical option to provide hemodynamic support in patients with RV failure is peripheral VA-ECMO, thus reducing RV preload. Veno-venous-ECMO (VV-ECMO) can deliver oxygenated blood to the RA, providing an oxyhemoglobin gradient across the interatrial septal defect to the left atrium to improve oxygenation and, possibly, hemodynamics (11). Rosenzweig et al. described a large retrospective cohort of 98 patients with highly heterogeneous pulmonary hypertension (groups 1, 3, 4, or 5) on ECLS (12). In 19 patients, ECLS was set up for a “bridge-to-recovery” strategy. Among them, only six (31.6%) survived hospital discharge. ECLS configuration was mostly set up in VA-ECMO (68%). However, VV-ECMO was set up in patients with adequate RV function and severe hypoxemia. In this case, VV-ECMO was initiated after mechanical ventilation. However, it is well described that positive pressure ventilation may increase right ventricle afterload and decrease right ventricle preload, leading to potential haemodynamic collapse (13). Then, in patients with PAH, VV-ECMO should be considered while spontaneous ventilation is allowed. One of the limitations of using ECMO in this situation is the introduction of anticoagulants. In cases of advanced heart failure, the benefit–risk balance must be considered, taking into account the advantages and limitations of ECMO.

In this study, severe hypoxemia was explained by a large RL shunt (estimation of 27%) through PFO, which was explained by a large increase in RV preload pressure.

### PAH drugs under ECLS

Optimal modalities of pharmacological treatment in patients with incident cases of PAH under ECLS are unknown. Currently approved therapies for PAH consist of endothelin receptor antagonists, phosphodiesterase type 5 inhibitors, and prostacyclin agents, which can be used in monotherapy, dual therapy, or triple therapy. Therapeutic use of intravenous prostanoids is associated with immediate vasodilatory action in the pulmonary circulation through action on vascular smooth muscle cells. However, antiproliferative and anti-inflammatory actions may be efficient only after a few days of effective treatment (14). Guidelines for treatment strategy for PAH are based on both baseline assessment and treatment response (9). The overall treatment goal for patients with PAH is to achieve a low-risk status associated with low-risk mortality. Initial triple combination therapy with intravenous prostacyclin in patients with severe PAH is now to be considered since studies revealed evidence of long-term benefits (9, 15, 16).

### Lung recovery or lung transplantation

In patients with PAH, lung transplantation should be considered soon after an inadequate clinical response to maximal medical therapy. In France since 2007, incident cases of PAH can be eligible for high emergency lung transplantation in case of WHO functional class IV associated with poor hemodynamic values (RVP greater than 20 WU and CI lower than 2 L/min/m^2^) under ECLS or mechanical ventilation despite a maximal combination therapy.

### PAH during labor

The incidence of the diagnosis of PAH associated with an initial severe form is unknown but may occur during pregnancy or during the post-partum period. Indeed, the risk of right heart failure is particularly high during labor, delivery, and the post-partum period, reflecting the high risks related to pressure and volume changes that occur during these stages (17). Interestingly, in our case, there was no evidence of right heart failure during her pregnancies, the last of which took place 36 months before her admission in the ICU.

## Conclusion

In conclusion, we report an incident case of heritable PAH revealed by refractory hypoxemia in which VA-ECMO can be used as an effective “bridge-to-recovery” strategy with prompt initiation of triple therapy and a dramatic hemodynamic and clinical improvement. More studies are needed to assess the benefits, timing, and limitations of ECLS as a “bridge-to-recovery” strategy.

## Data availability statement

The original contributions presented in the study are included in the article, further inquiries can be directed to the corresponding author.

## Ethics statement

Ethical approval was not required for the studies involving humans because the patient has given the right to use her data. The studies were conducted in accordance with the local legislation and institutional requirements. The human samples used in this study were acquired from a by-product of routine care or industry. Written informed consent to participate in this study was not required from the participants or the participants’ legal guardians/next of kin in accordance with the national legislation and the institutional requirements. Written informed consent was obtained from the individual(s) for the publication of any potentially identifiable data included in this article. Written informed consent was obtained from the participant/patient(s) for the publication of this case report.

## Author contributions

BP: Conceptualization, Formal analysis, Investigation, Methodology, Project administration, Resources, Software, Supervision, Validation, Visualization, Writing – original draft, Writing – review & editing. AC: Methodology, Resources, Supervision, Validation, Writing – review & editing. FC: Conceptualization, Methodology, Supervision, Validation, Writing – review & editing. BL: Conceptualization, Investigation, Methodology, Supervision, Validation, Writing – review & editing. SV: Conceptualization, Data curation, Investigation, Methodology, Project administration, Supervision, Validation, Visualization, Writing – original draft, Writing – review & editing.

## Figures and Tables

**Table:** 

6MWD	6-min walk test distance
BMPR2	Bone morphogenetic protein receptor 2
CT	Computed tomography
DL_CO_	Diffusing capacity of the lung for carbon monoxide
dPAP	Diastolic pulmonary arterial pressure
ECLS	Extra corporeal life support
F_I_O_2_	Fraction of inspired oxygen
HFNC	High flow nasal cannula
ICU	Intensive care unit
mPAP	Mean pulmonary arterial pressure
NT-proBNP	N-terminal prohormone of brain natriuretic peptide
PAH	Pulmonary arterial hypertension
P_A_O_2_	Alveolar partial pressure of oxygen
PaO_2_	Arterial partial pressure of oxygen
PCWP	Pulmonary-capillary wedge pressure
PFO	Patent foramen ovale
PVR	Pulmonary vascular resistance
RAP	Right atrial pressure
RV	Right ventricle
sPAP	Systolic pulmonary arterial pressure
TTE	Transthoracic echocardiogram
VA-ECMO	Venoarterial extracorporeal membrane oxygen
VV-ECMO	Venovenous extracorporeal membrane oxygen
WHO	World Health Organization
WU	Wood unit
RL	Right-to-left
ABG	Arterial blood gas
